# Sniffing Out Chemosensory Genes from the Mediterranean Fruit Fly, *Ceratitis capitata*

**DOI:** 10.1371/journal.pone.0085523

**Published:** 2014-01-08

**Authors:** Paolo Siciliano, Francesca Scolari, Ludvik M. Gomulski, Marco Falchetto, Mosè Manni, Paolo Gabrieli, Linda M. Field, Jing-Jiang Zhou, Giuliano Gasperi, Anna R. Malacrida

**Affiliations:** 1 Department of Biology and Biotechnology, University of Pavia, Pavia, Italy; 2 Department of Biological Chemistry and Crop Protection, Rothamsted Research, Harpenden, United Kingdom; Virginia Tech, United States of America

## Abstract

The Mediterranean fruit fly, *Ceratitis capitata* (medfly), is an extremely invasive agricultural pest due to its extremely wide host range and its ability to adapt to a broad range of climatic conditions and habitats. Chemosensory behaviour plays an important role in many crucial stages in the life of this insect, such as the detection of pheromone cues during mate pursuit and odorants during host plant localisation. Thus, the analysis of the chemosensory gene repertoire is an important step for the interpretation of the biology of this species and consequently its invasive potential. Moreover, these genes may represent ideal targets for the development of novel, effective control methods and pest population monitoring systems. Expressed sequence tag libraries from *C. capitata* adult heads, embryos, male accessory glands and testes were screened for sequences encoding putative odorant binding proteins (OBPs). A total of seventeen putative *OBP* transcripts were identified, corresponding to 13 Classic, three Minus-C and one Plus-C subfamily OBPs. The tissue distributions of the *OBP* transcripts were assessed by RT-PCR and a subset of five genes with predicted proteins sharing high sequence similarities and close phylogenetic affinities to *Drosophila melanogaster* pheromone binding protein related proteins (PBPRPs) were characterised in greater detail. Real Time quantitative PCR was used to assess the effects of maturation, mating and time of day on the transcript abundances of the putative *PBPRP* genes in the principal olfactory organs, the antennae, in males and females. The results of the present study have facilitated the annotation of *OBP* genes in the recently released medfly genome sequence and represent a significant contribution to the characterisation of the medfly chemosensory repertoire. The identification of these medfly *OBP*s/*PBPRP*s permitted evolutionary and functional comparisons with homologous sequences from other tephritids of the genera *Bactrocera* and *Rhagoletis*.

## Introduction

The Mediterranean fruit fly, *Ceratitis capitata*, is a serious agricultural pest that has expanded from its native range in East Africa to attain an almost worldwide distribution. Its biological success and invasive potential are due to its ability to readily adapt to new environments, to complete multiple generations each year utilising different host plants as they become available, and its high reproductive capacity [1]. Unlike *Drosophila* species that inhabit and feed on rotting and decaying organic material, the medfly has evolved an opportunistic phytophagous lifestyle [2], [3]. These very different food resource exploitation strategies would be expected to be reflected in adaptive differences in the abilities of these two species to detect, and respond to, different plant volatiles and odours.

Insect chemoreception is facilitated by a signal transduction cascade involving three main groups of molecules, odorant-binding proteins (OBPs) [4], [5], chemosensory proteins (CSPs) [6], and the chemoreceptor superfamily formed by the olfactory (OR), gustatory (GR) and ionotropic (IR) receptor families [5]. Insect OBPs are small, globular, abundant water-soluble proteins, characterised by a domain of six α-helices, joined by either two or three disulphide bonds [7], [8], that are secreted into the sensillar lymph by non-neuronal auxiliary cells. Odorant molecules that enter the pores in the sensilla are bound and solubilized by OBPs and transported through the aqueous lymph to activate the membrane bound ORs [5], [9], [10]. The *Drosophila* OBP gene family has been divided into a number of subfamilies, defined on distinctive structural and functional features and phylogenetic relationships (Classic, Minus-C, Plus-C, Dimer, PBP/GOBP, ABPI and ABPII, CRLBP, and D7 subfamilies)[10]–[16]. In *Drosophila* OBPs have been shown to be implicated in the recognition of the male courtship pheromone [17], [18] and host-plant selection [19]. However, not all OBPs are restricted to chemosensory tissues and may participate in other physiological functions [6], [20]–[23].

Chemoreception plays an important role in medfly courtship behaviour. The mating system is based on arboreal aggregations (leks) of sexually mature males [24]–[27]. The males actively defend favoured positions in the lek and emit a sex pheromone from their everted rectal ampulla which is both attractive to females and able to ‘call’ other males to the lek site [24], [28]. When a receptive female approaches, the male vibrates his wings in a continuous manner, apparently wafting a plume of pheromone towards the female [29]. The components of the pheromone mixture emitted by the male have been identified [30]–[34]. Medfly females use a different pheromone to mark fruit after oviposition that acts as a deterrent to further egg-laying [2].

Despite the evident importance of plant volatiles and pheromones in medfly behaviour, little is known about the chemosensory proteins involved in their detection [35], [36]. Here we report the identification of a number of OBP transcripts. We used EST libraries [23], [35] derived from adult heads as these include the main olfactory organs of the medfly, from the male reproductive tract as studies have shown that OBPs are expressed in such tissues in other insects [37]–[39], and from embryos, which, being enriched for late embryonic stages, could provide sequences involved in larval perception during their development in the fruit. A subset of the identified OBPs that shared the highest similarities with genes encoding putative pheromone binding proteins (PBPs) were characterised in greater detail both at the molecular and physiological levels. With the recent release of the medfly genome within the i5K initiative (http://arthropodgenomes.org/wiki/Ceratitis_capitata), our analyses represent a significant contribution to the annotation of the entire medfly chemosensory repertoire.

Moreover, unraveling the molecular machinery of chemoreception in the medfly is the basis for the development of innovative, environmentally-friendly, pest control strategies against this species.

## Materials and Methods

### General Approach

The experimental approach used in this paper included: (i) identification of putative *OBP* transcripts from three EST libraries constructed from embryo, male/female head, testes/male accessory glands [23], [35] and their annotation in the recently released medfly genome; (ii) assessment of *OBP* transcript tissue-specificity and phylogenetic analyses of their predicted proteins; (iii) genetic characterisation of a subset of *OBP* genes that may represent *PBP*-related protein (*PBPRP*) candidates; (iv) assessment of the relative transcript abundances of these candidate genes in the main olfactory organs of each sex; (v) analysis of the effects of maturation/mating/time of day on transcript abundances in the antennae of each sex.

### *OBP* Identification and Annotation from Medfly EST Libraries

BLASTX searches (*e*-value threshold of 10^−5^) were performed using the NCBI server [40] to identify putative *OBP* transcript sequences present in three medfly EST libraries [23], [35]. The transcripts were then reassembled using CAP3 to identify redundancy between the libraries [41]. Putative ORFs and associated amino acid sequences were determined using CLC Main Workbench version 6.6. These candidates were analysed for the presence of all the OBP hallmarks, namely the presence of signal peptide sequences, using the SignalP 4.0 server [42], and the presence of the characteristic conserved cysteine residues [6], [14].

### Medfly Samples

Virgin and mated adult individuals of both sexes from the established medfly ISPRA strain were used in this study. Standard rearing methods were employed [43]; these included a photoperiod of L12: D12 with photophase starting at 08∶00 h and a constant temperature of 24°C and 60% humidity. For the analysis of the tissue-specificity of the *OBP* transcripts, four day-old (4d) sexually mature individuals were used. For the assessment of the *OBP* transcriptional changes potentially induced by maturation and/or mating, one day-old (1d) immature and four day-old (4d) sexually mature virgin/mated individuals were collected. To obtain mated flies, approximately two-hundred 4d virgin flies of each sex were introduced into a 25×25×25 cm cage shortly after the beginning of the photophase. As copulating pairs formed, they were collected in small vials and removed from the cage. Only pairs that maintained *copula* for at least 100 minutes were used in order to avoid false matings, i.e. those in which little or no sperm are transferred [44].

### Reverse Transcriptase-PCR (RT-PCR) for the Analysis of the Tissue-specificity of *OBP* Transcripts

Total RNA was extracted from different body parts of virgin mature male and female flies using Trizol, according to the manufacturer’s instructions. Pools of each of the following body parts were used: antennae (∼150 pairs), palps (∼150 pairs), heads without antennae and palps (5), tarsi (∼60 sets), legs without tarsi (∼60 sets), thoraces without wings and legs (5), abdomens (5) and wings (∼75 pairs). After DNAse treatment (DNAfree, Ambion), RNA integrity was determined by formaldehyde agarose gel electrophoresis and quantified using a Nanodrop ND-1000 spectrophotometer (Nanodrop Technologies Inc., Wilmington, DE, USA). For each body part 200 ng of the extracted total RNA was transcribed into cDNA using the iScript™ cDNA Synthesis Kit (Biorad). RT-PCRs with gene specific primers, designed using Beacon Designer 7 (Premier Biosoft International) (Table S1), were performed using 5% of the synthesized cDNA and the following cycle conditions: 94°C for 3 minutes, 30 cycles at 94°C for 30 seconds, 57°C for 30 seconds, 72°C for 2 minutes, and a final extension at 72°C for 10 minutes. The medfly *GAPDH2* reference gene was amplified as a control for cDNA integrity. To control for genomic DNA contamination, RT-PCR was also performed on samples in which cDNA synthesis had been performed in the absence of reverse transcriptase. The amplification products were electrophoresed on 2% agarose gels.

### Phylogenetic Analyses

A phylogenetic analysis was performed including the medfly OBP predicted amino acid sequences and the 52 known *D. melanogaster* OBPs [10], [11], [15]. After excluding the signal peptide sequences [10], the amino acid sequences were aligned using MAFFT v6.935b [45] with the E-INS-i strategy, BLOSUM62 matrix, 1000 maxiterate and offset 0. The most appropriate model of molecular evolution for the dataset was determined using MEGA 5.2.2 [46]. Phylogenetic relationships were estimated using Maximum Likelihood with 1000 bootstrap replications using MEGA 5.2.2. The resulting mid-point rooted tree was drawn using the FigTree v1.4 (http://tree.bio.ed.ac.uk/software/figtree/).

A phylogenetic analysis was also performed including the medfly OBP amino acid sequences and the available putative OBPs from three other tephritid species: the Oriental fruit fly *Bactrocera dorsalis s.s.*, the Northern walnut husk fly *Rhagoletis suavis*, and the apple maggot *Rhagoletis pomonella*. Specifically, we considered: ten OBPs from *B. dorsalis* [47] and BdorOBP2 (unpublished, GenBank accession no. AGO28153), nine OBPs from *R. suavis* [48], and fifteen OBPs from *R. pomonella* [49]. For *R. pomonella* and *R. suavis*, we determined the OBP amino acid sequences using CLC Main Workbench version 6.6. We also renamed the *R. pomonella* OBPs based on their similarities with *D. melanogaster* homologues (Table S2). The phylogenetic analysis was performed as described above.

### Characterisation of Gene Structure of the Putative Medfly Pheromone Binding Protein (*PBP*) Genes

A subset of medfly *OBP* genes was selected for exon-intron structure characterization, on the basis of their transcriptional tissue-specificity in the main olfactory organs and their sequence similarity to *Drosophila PBPRP* genes. The 5′ and 3′ ends of the transcripts were identified by rapid amplification of cDNA ends (RACE), using RNA extracted from the heads of 4d mature virgin males and females, with the GeneRacer™ Kit (Life Technologies). Primers (Table S3) were designed on the medfly transcript sequences using PRIMER3 [50]. PCR products were cloned into pCR®4-TOPO vector (Life Technologies) and sequenced.

Introns and exons of the medfly putative *PBPRP* genes were identified by comparing the transcripts with the genomic sequences obtained by PCR amplification of pooled male/female genomic DNA [51]. In each reaction, 10 ng genomic DNA, 1.5 mM MgCl_2_, Reaction buffer (10 mM Tris, 50 mM KCl; pH 8.3), 0.2 mM dNTPs, 10 pmol of each primer and 1 unit *Taq* DNA polymerase (Life Technologies) were used, with the following cycle conditions: 94°C for 3 minutes, 30 cycles at 94°C for 45 seconds, 57–60°C for 30 seconds, 72°C for 2 minutes, and a final extension at 72°C for 10 minutes. PCR products were analysed by 1.0% agarose-gel electrophoresis, purified using the High Pure PCR Product Purification kit (Roche), cloned and sequenced. Sequences were analysed using CLC Main Workbench 6.6 and EST2GENOME [52].

### Real-Time Quantitative PCR

The cDNAs derived from antennae, palps and tarsi of mature virgin (4d) male and female flies were used to assess the relative transcript abundance of the putative *PBPRP* genes in these organs. Two medfly reference genes (*GAPDH2* and *G6PDH*) were used for normalization [53]. Real Time qPCR with specific primers (Table S1) was performed using SsoFast™ EvaGreen® Supermix (Biorad) and 5% of the synthesized cDNA on a MiniOpticon (Biorad). Cycling parameters were: 3 minutes at 95°C, 40 cycles of 10 seconds at 95°C and 30 seconds at 55°C and 30 seconds at 68°C. Fluorescence was detected at the end of each extension step. Three technical replicates were performed and the specificity of the amplification products was assessed by melt-curve analysis. PCR efficiencies were above 90% for all primer pairs. Data analysis was performed using CFX Manager Software, Version 1.5 (Biorad) and unpaired 2-tailed t-tests with Sidàk’s correction for multiple comparisons [54].

Real-Time qPCR was also used to assess the effect of sexual maturation and mating on the transcriptional profiles of the putative medfly *PBPRP* genes in the antennae. For this, total RNA was extracted from the antennae (∼150 pairs) of immature (1d) and mated (4d) male and female flies as described previously. The cDNAs derived from these samples, together with those from the mature virgin (4d) male and female antennae, were used for Real-Time qPCR as described previously. The immature virgin male samples were taken as calibrators in order to assess the relative fold-change during maturation and after mating.

For the analysis of the effect of time of day on transcript abundance, Real Time qPCR was performed on cDNA derived separately from the antennae of mature virgin (4d) males and females, harvested between 09∶00 and 11∶00 hrs (1 to 3 hrs after the beginning of the photophase). These cDNAs were compared to the mature virgin male and female antennal cDNAs derived from RNA harvested between 14∶00 and 16∶00 hrs (6 to 8 hrs after the beginning of the photophase). These two time points were chosen to cover the morning and early afternoon periods of peak sexual activity [55] as confirmed in our insectary conditions. The mature virgin male samples (09∶00–11∶00 hrs) were taken as calibrators in order to assess the relative fold-change.

## Results

### Identification of Medfly Putative Odorant Binding Protein Transcripts

BLAST analyses indicated that a total of 51 assembled transcripts from the three medfly libraries shared similarities with insect *OBP* genes. Two of these were derived from the embryo, 28 from the adult head and 21 from the testes/male accessory gland libraries [23], [35]. After the removal of redundant sequences among the three libraries, 17 unique *OBP* gene transcripts were identified. BLASTX analyses against the *Drosophila* peptide database allowed us to assess their identity/similarity with their putative *Drosophila* homologues (Table 1). On this basis, the 17 medfly *OBP* genes were provisionally named after their putative *Drosophila* homologues. Transcripts which shared highest similarity to the same *Drosophila* OBP were differentiated with a numerical postscript.

**Table 1:** Medfly assembled sequences that share significant similarity to odorant binding protein genes.

		BLASTX against *D. melanogaster* peptide database							TBLASTX against *C. capitata* genome predicted peptide database		
*OBP*	Library	Best hit	*e*-value	Identity/Similarity (%)	Predicted amino acids	Conserved cysteine spacing	Signal peptide (position, D-score)	Subfamily	Best hit	*e*-value	Identity/Similarity (%)
*CcapOBP8a*	Embryo	OBP8a	2e-16	32/51	162	30-38-12-5	28, 0.757	Minus-C^1^	XM_004521128.1	3e-108	100/100
*CcapOBP19a*	Head	OBP19a	1e-49	60/78	147	26-3-40-10-8	26, 0.668	Classic	XM_004524969.1	2e-98	100/100
*CcapOBP19b*	Head	OBP19b	1e-29	38/58	151	24-3-41-12-8	19, 0.829	Classic	XM_004524970.1	2e-103	98/98
*CcapOBP19d-1*	Head	OBP19d/PBPRP2	1e-24	43/65	142	26-3-43-9-8	20, 0.719	Classic	XM_004524978.1	2e-95	100/100
*CcapOBP19d-2*	Head	OBP19d/PBPRP2	6e-14	33/55	143	26-3-43-9-8	20, 0.875	Classic	XM_004525083.1	1e-70	99/99
*CcapOBP28a*	Head	OBP28a/PBPRP5	3e-33	50/65	147	26-3-43-9-8	20, 0.821	Classic	XM_004524959.1	1e-100	100/100
*CcapOBP44a*	Head, TAG	OBP44a	6e-52	62/77	142	26-3-39-10-8	17, 0.877	Classic	XM_004535885.1	6e-96	100/100
*CcapOBP49a*	TAG	OBP49a	1e-01	40/60	>127	–	–	–	XM_004522926.1	3e-86	100/100
*CcapOBP56d*	Head, TAG	OBP56d	8e-27	41/64	137	27-3-37-8-8	18, 0.903	Classic	XM_004517746.1	7e-77	100/100
*CcapOBP56h*	Head	OBP56h	6e-19	35/56	124	27-3-34-8-8	19, 0.861	Classic	XM_004518409.1	7e-72	99/99
*CcapOBP69a*	Head	OBP69a/PBPRP1	1e-27	37/62	147	27-3-37-8-8	23, 0.753	Classic			
*CcapOBP83a-1*	Head	OBP83a/PBPRP3	4e-77	69/79	157	26-3-37-8-8	33, 0.844	Classic	XM_004523388.1	4e-111	100/100
*CcapOBP83a-2*	Head	OBP83a/PBPRP3	3e-52	55/70	148	26-3-37-8-8	23, 0.901	Classic	XM_004523387.1	3e-103	99/99
*CcapOBP84a-1*	Embryo, Head	OBP84a/PBPRP4	7e-29	45/62	177	29-3-32-10-8	22, 0.822	Classic	XM_004529312.1	2e-77	100/100
*CcapOBP84a-2*	Head	OBP84a/PBPRP4	3e-34	51/71	174	29-3-32-8-8	26, 0.726	Classic			
*CcapOBP99c*	TAG	OBP99c	4e-52	58/79	149	30-38-19	16, 0.877	Minus-C	XM_004521129.1	2e-91	100/100
*CcapOBP99d*	TAG	OBP99d	3e-23	46/65	151	24-38-18	19, 0.803	Minus-C	XM_004521127.1	1e-96	97/98

^1^ Lacks C2.

The predicted translations of the complete medfly *OBP* transcripts ranged from 124 (*CcapOBP56h*) to 177 amino acids (*CcapOBP84a-1*). The *CcapOBP49a* transcript was clearly truncated at the 5′ end and hence it was not possible to determine the length of its polypeptide. All of the other transcripts encoded polypeptides with signal peptides. On the basis of the conserved cysteine profiles, 13 had the six conserved cysteine residues typical of Classic OBPs [11], [56]. Three, *CcapOBP8a*, *CcapOBP99c* and *CcapOBP99d,* encoded putative polypeptides with four or five (*CcapOBP8a*) conserved cysteine residues, and thus represent Minus-C OBPs [11]. The availability of these 17 *OBP* assembled transcripts allowed us to annotate eleven medfly genomic sequences recently released in the NCBI as Classic OBPs, and three as Minus-C OBPs (Table 1). The medfly genome also contains the full length sequence (XM_004522926.1) of the truncated *CcapOBP49a* transcript identified in the TAG library. This full length copy encodes a 253 amino acid polypeptide that belongs to the Plus-C OBP subfamily, with a conserved cysteine spacing of 0-19-17-11-3-43-20-9-8-10. Compared to *D. melanogaster*, the polypeptide derived from the truncated *CcapOBP49a* transcript shared low amino acid similarity with the Plus-C OBP. Interestingly, the CcapOBP49a full length sequence displays 35/53% amino acid identity/similarity (*e* = 7e-32) with its *Ae. aegypti* orthologue AaegOBP23.

The medfly *OBP* genes are available in GenBank with the accession numbers reported in Table 1. The sequences of *CcapOBP69a* and *CcapOBP83a-2* have been deposited in the European Nucleotide Archive (ENA) under accession numbers HG764550 and HG764551, respectively.

### Tissue- and Sex-specificity of the Medfly *OBP* Transcripts

**Figure 1:**
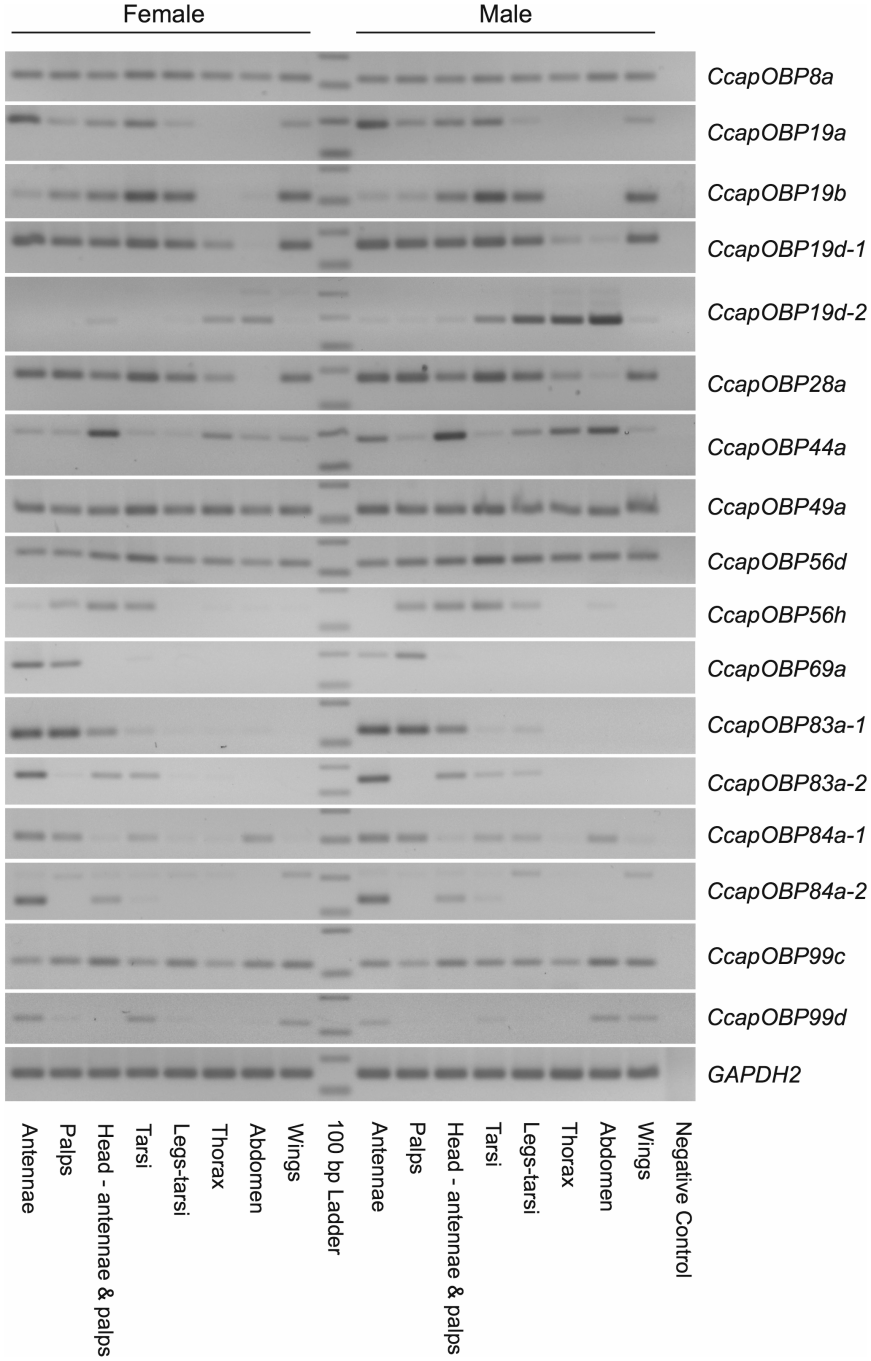
Transcriptional profiles of the *C. capitata OBP* genes in different body parts of 4 day-old virgin males and females as determined by RT-PCR.

The identified medfly *OBP* transcripts display different patterns of tissue distribution and abundance (Figure 1). The transcripts of *CcapOBP8a*, *CcapOBP49a*, *CcapOBP56d, CcapOBP99c*, and *CcapOBP44a* are present in all body parts in both sexes. Among these, the *CcapOBP44a* transcript appears to be particularly abundant in the head. The transcripts of *CcapOBP69a*, *CcapOBP83a-1*, *CcapOBP83a-2, CcapOBP84a-1* and *CcapOBP84a-2* are present predominantly in the main olfactory organs, i.e. antennae and/or maxillary palps (Figure 1; Figure S1). *CcapOBP99d* is abundant in the antennae, but also present in tarsi, wings and male abdomen.

### Phylogenetic Analysis of the Medfly and *Drosophila* OBPs

**Figure 2:**
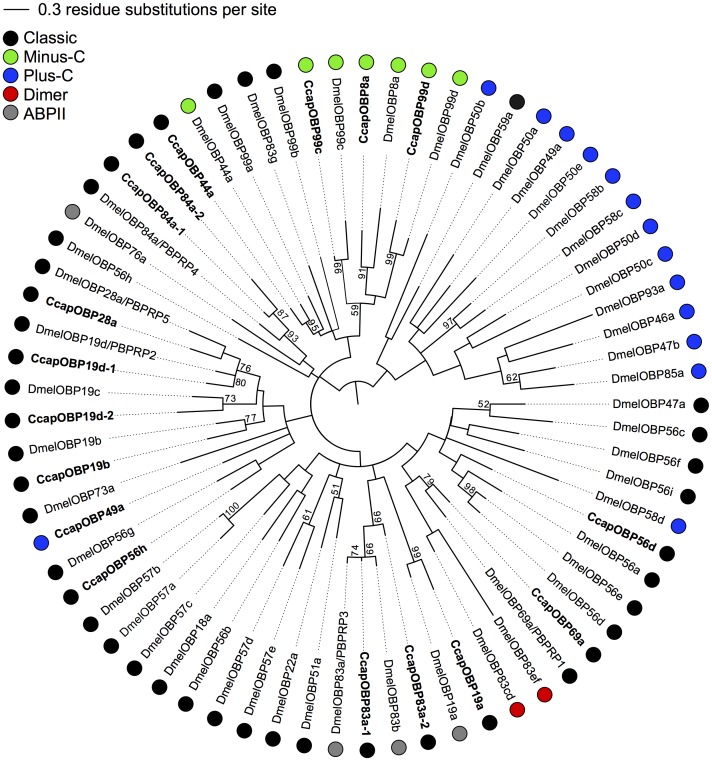
Phylogenetic relationships of OBP proteins from *C. capitata* and *D. melanogaster*. The unrooted maximum-likelihood (log likelihood = −6336.82) tree was inferred using the Whelan and Goldman model [85] and a discrete Gamma distribution. Bootstrap values greater than 50% (1000 replications) are shown. Coloured circles indicate the different OBP subfamilies.

The phylogenetic relationships of the 17 putative medfly OBPs and the 52 *D. melanogaster* OBPs, as well as their classification into different subfamilies [10], are shown in the Maximum Likelihood mid-point rooted tree in Figure 2. Bootstrap support for deeper branches was generally weaker than for terminal branches and branch lengths vary considerably within each clade. The global tree topology suggests that the medfly OBPs are not clustered in species-specific clades, they generally tend to be grouped with the *D. melanogaster* OBP that produced the best BLASTX hit (Table 1). The three medfly Minus-C OBPs, CcapOBP8a, CcapOBP99c, and CcapOBP99d, cluster together with their corresponding *D. melanogaster* Minus-C orthologues. This grouping is further supported by the spacing pattern of conserved cysteines (Table 1). All the other medfly OBPs, which were assigned to the Classic subfamily, are spread in different clades with the *Drosophila* Classic OBPs.

Interestingly, seven of the medfly Classic OBPs share consistent phylogenetic relationships with the *Drosophila* OBPs which have been classified as PBPRPs [57], as supported by high bootstrap values at the terminal branches. In fact, CcapOBP69a clusters with DmelOBP69a/PBPRP1; CcapOBP83a-1 and CcapOBP83a-2, which share 51/75% identity/similarity, are tightly related to DmelOBP83a/PBPRP3; CcapOBP19d-1 and CcapOBP28a are grouped with DmelOBP19d/PBPRP2 and DmelOBP28a/PBPRP5; CcapOBP84a-1 and CcapOBP84a-2, which share 41/58% identity/similarity, are most closely related to DmelOBP84a/PBPRP4.

### Phylogenetic Analysis of OBPs from *C. capitata*, *B. dorsalis*, *R. suavis* and *R. pomonella*

**Figure 3:**
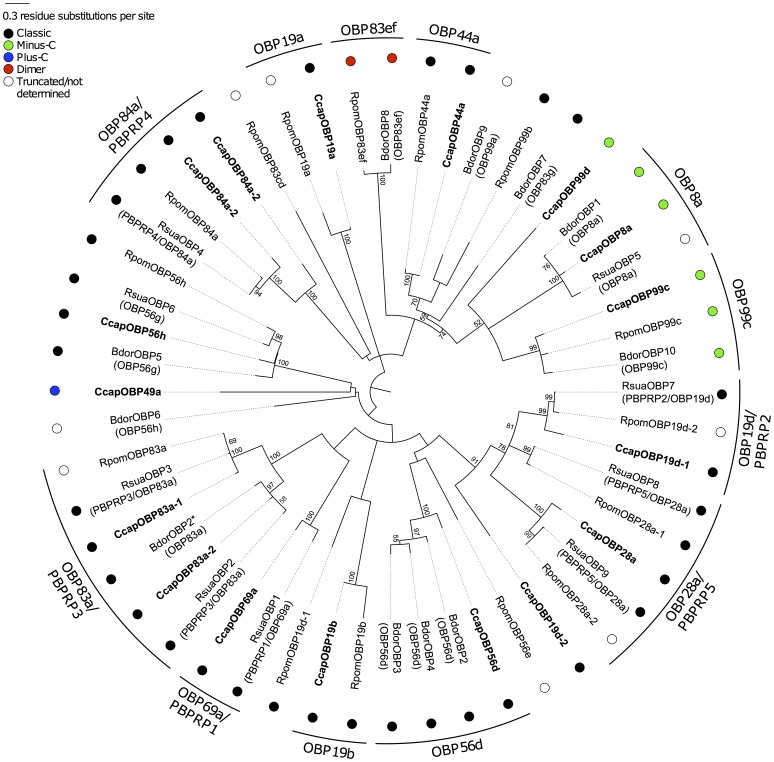
Phylogenetic relationships of tephritid OBP proteins. The unrooted maximum-likelihood (log likelihood = −9096.49) tree was inferred using the Whelan and Goldman model [85] and a discrete Gamma distribution and some invariable sites. Bootstrap values greater than 50% (1000 replications) are shown. Coloured circles indicate the different OBP subfamilies.

The Maximum Likelihood mid-point rooted tree in Figure 3 portrays the phylogenetic relationships among the 17 *C. capitata* OBPs and those available from *B. dorsalis* (eleven OBPs), *R. suavis* (nine OBPs) and *R. pomonella* (fifteen OBPs), as well as their classification into different subfamilies [10]. As expected, each medfly OBP is clustered together with the OBPs from the other tephritid species that produced the best BLASTP hits (Table S4). The Minus-C OBPs from the different species cluster together, whereas the medfly Plus-C CcapOBP49a shares no significant similarity with any of the available tephritid sequences (Table S4). Interestingly, the seven medfly PBPRPs (CcapOBP69a, CcapOBP83a-1, CcapOBP83a-2, CcapOBP19d-1, CcapOBP28a, CcapOBP84a-1 and CcapOBP84a-2) are distributed in five well distinct clades together with sequences from the two *Rhagoletis* species. Each of these five clusters include sequences sharing high similarity to the *Drosophila* DmelOBP69a/PBPRP1, DmelOBP83a/PBPRP3, DmelOBP19d/PBPRP2, DmelOBP28a/PBPRP5, and DmelOBP84a/PBPRP4 (Figure 3). The only *B. dorsalis* OBP that clusters within a PBPRP clade is BdorOBP2(OBP83a).

### Gene Structure of the Medfly Putative *PBPRP*s

*CcapOBP19d-1*, *CcapOBP28a*, *CcapOBP69a*, *CcapOBP83a-1* and *CcapOBP83a-2* were further characterized in terms of gene structure and compared to their *Drosophila* counterparts. *CcapOBP84a-1* and *CcapOBP84a-2* were not considered due to their high correlation with *DmelOBP84a/Pbprp4* which, in spite of being classified as a Classic OBP, is the OBP most related to the highly heterogeneous Plus-C subfamily [11], [13], [58].

**Figure 4:**
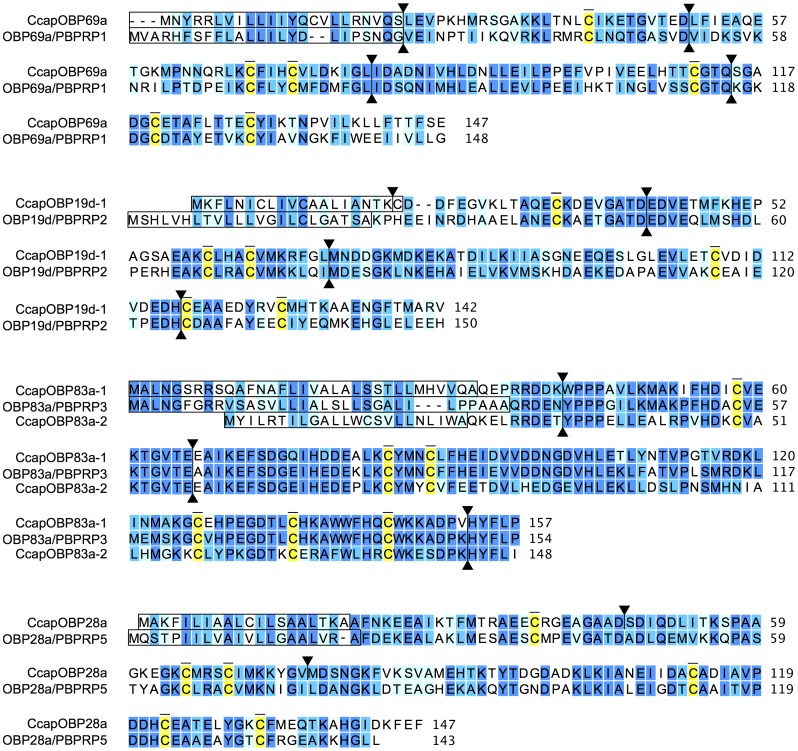
Alignments of the predicted amino acid sequences of five *C. capitata* OBPs with their putative *D. melanogaster* orthologues. Identical amino acids are shown on a dark blue background, medium and light blue backgrounds indicate positions with strongly and weakly similar properties, respectively. Conserved cysteine residues are highlighted in yellow. The signal peptide sequences are boxed. The positions of introns are indicated by triangles.

The complete transcript sequences were obtained for each of the candidate medfly genes using RACE PCR, resulting in full-length transcripts ranging from 698 to 883 bp in length. For each transcript, a coding sequence was identified that ranged from 429 to 474 bp. Figure 4 shows the alignments between the predicted amino acid sequences of these five medfly putative PBPRPs and their *Drosophila* counterparts. The sequence identity/similarity varies from 32.6%/56.7% (CcapOBP19d-1/OBP19d) and 68.2%/80.3% (CcapOBP83a-1/OBP83a).

Comparison of exon/intron structure indicates that the intron number and position are not highly conserved between medfly and *Drosophila*: *CcapOBP19d-1* has an extra intron compared to *DmelOBP19d*/*PBPRP2*; *CcapOBP83a-1* and *CcapOBP83a-2* share three introns with *DmelOBP83a*/*PBPRP3,* but the *Drosophila* orthologue contains an additional fourth intron in the 5′UTR; *CcapOBP28a* contains two introns that are absent in the intronless *DmelOBP28a*/*PBPRP5*. Only *CcapOBP69a* shares conserved intron number (four) and position with *DmelOBP69a*/*PBPRP1*. Introns were generally longer in medfly genes compared to their *Drosophila* orthologues.

### Transcriptional Profiles of the Putative Medfly *PBPRP* Genes in the Main Olfactory Tissues and in Relation to Sexual Maturation and Mating

**Figure 5:**
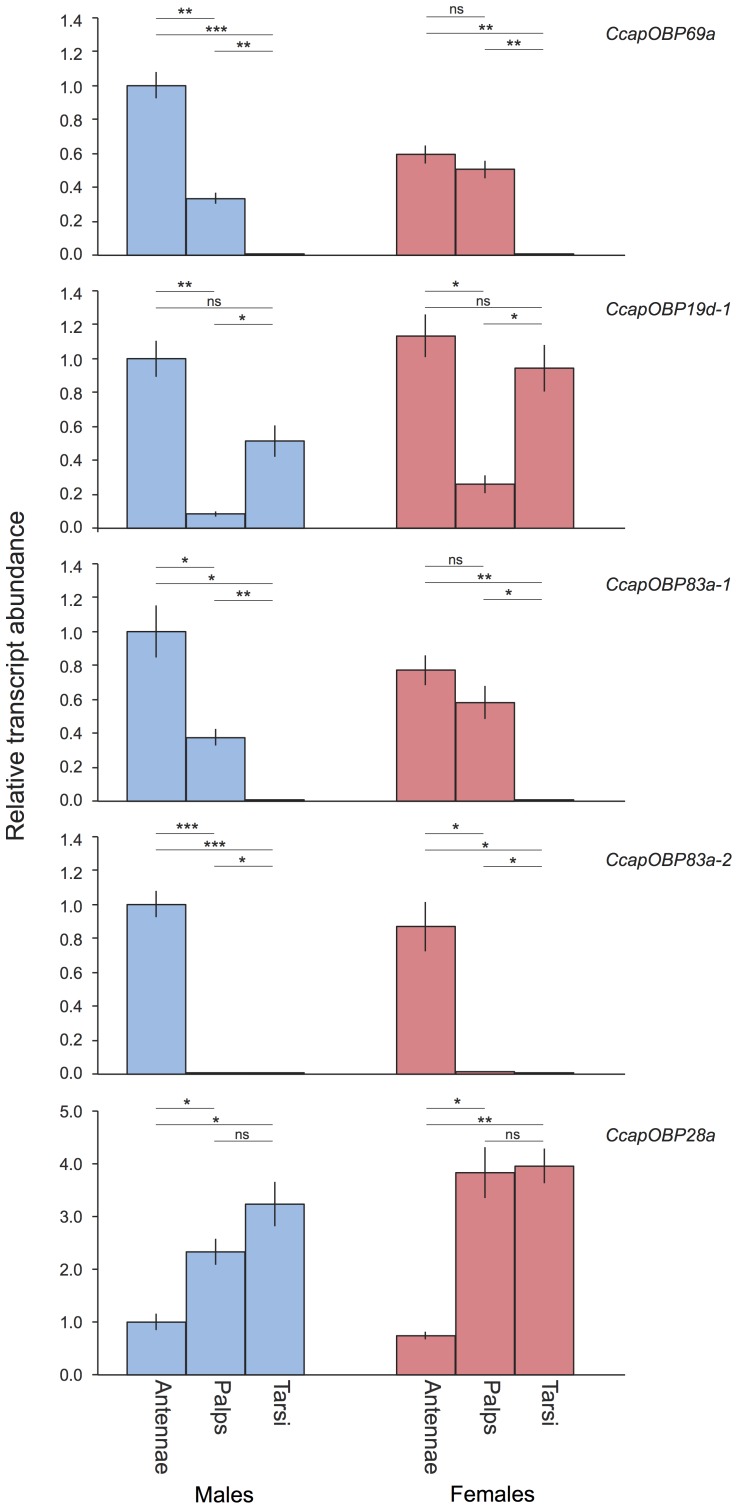
Transcript abundances of five *OBP* genes in the antennae, palps and tarsi of mature virgin males and females. Asterisks indicate significant differences in transcript abundances (*P<0.05, **P<0.01, ***P<0.001, unpaired 2-tailed t-tests with Sidàk’s correction for multiple comparisons).

The relative transcript abundances of the five putative medfly *PBPRP* genes in the antennae, maxillary palps and tarsi of virgin sexually mature males and females are shown in Figure 5. In both sexes, transcription is highest in the antennae for *CcapOBP69a, CcapOBP19d-1, CcapOBP83a-1* and *CcapOBP83a-2*. *CcapOBP83a-2* appears to be almost exclusively transcribed in the antennae, but the other three are also transcribed, at lower levels, in the palps (*CcapOBP69a, CcapOBP19d-1, CcapOBP83a-1*) and in the tarsi (*CcapOBP19d-1*), with relatively higher transcript abundance in the females.

By contrast, *CcapOBP28a* is present in the antennae, but is more abundant in the palps and tarsi. Its relative abundance in the male tarsi is 3-fold higher than in the antennae, whereas in the female both palps and tarsi exhibit 3.5-fold higher abundance than in antennae. Thus, this gene may have a biological role in all three tissues.

**Figure 6:**
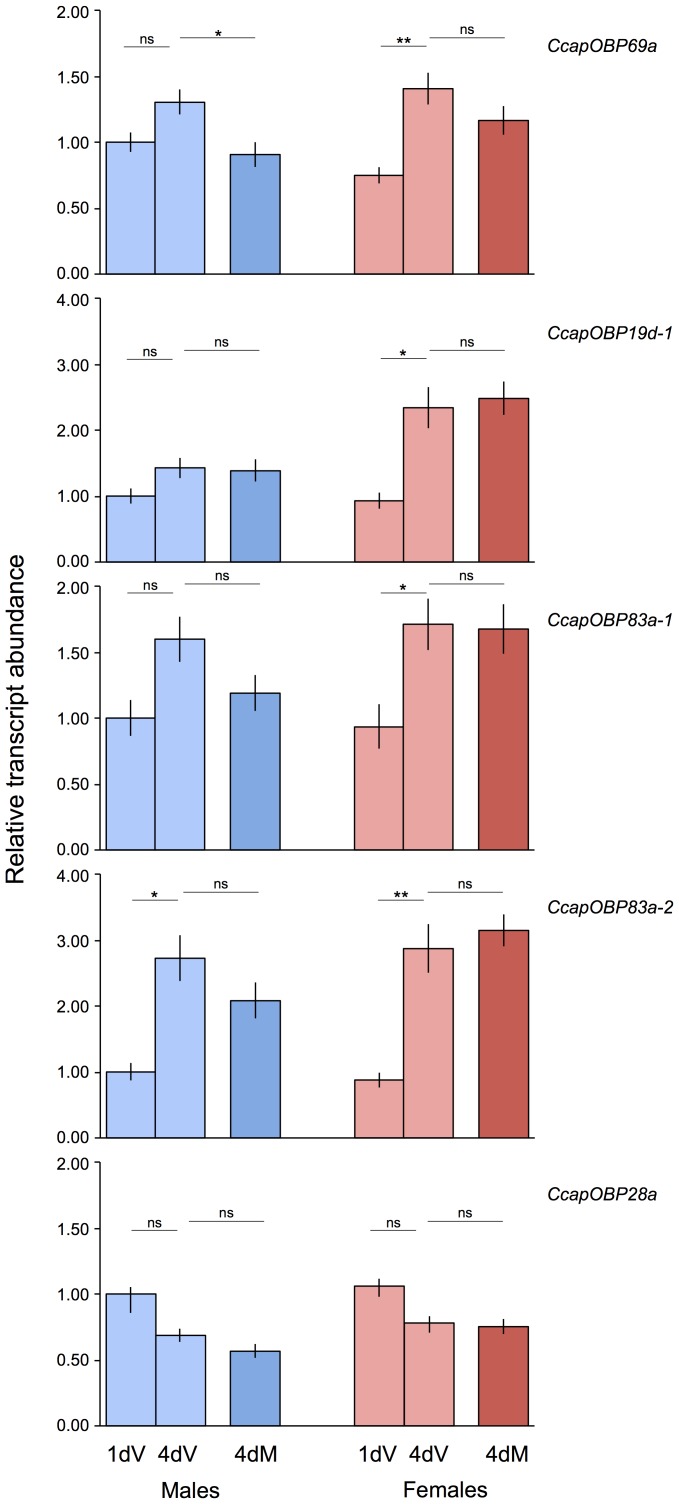
Transcript abundances of five *OBP* genes in the antennae of 1 day immature (1 dV), 4 day mature virgin (4 dV) and 4 day-old mated (4 dM) males and females. Asterisks indicate significant differences in transcript abundances (*P<0.05, **P<0.01, unpaired 2-tailed t-tests).

Considering that the antennae are known to be the main olfactory tissues in the medfly [59], we determined the impact of maturation and mating on the transcript abundances of the five putative *PBPRP* genes in the antennae. A trend of increasing transcript abundance is evident as a consequence of female maturation for *CcapOBP69a, CcapOBP19d-1, CcapOBP83a-1* (approximately 2-fold change) and *CcapOBP83a-2* (approximately 3-fold change)(Figure 6). Conversely, in males the only gene that changes during maturation is *CcapOBP83a-2* (unpaired t-test, *P*<0.05). None of the five genes appear to be modulated by mating in the females, whereas in the males there is a general trend of decreased transcriptional activity although this is significant only for *CcapOBP69a.* Finally, *CcapOBP28a* displayed a slight, but insignificant, reduction in transcript abundance related to maturation in both sexes and was not affected by mating.

**Figure 7:**
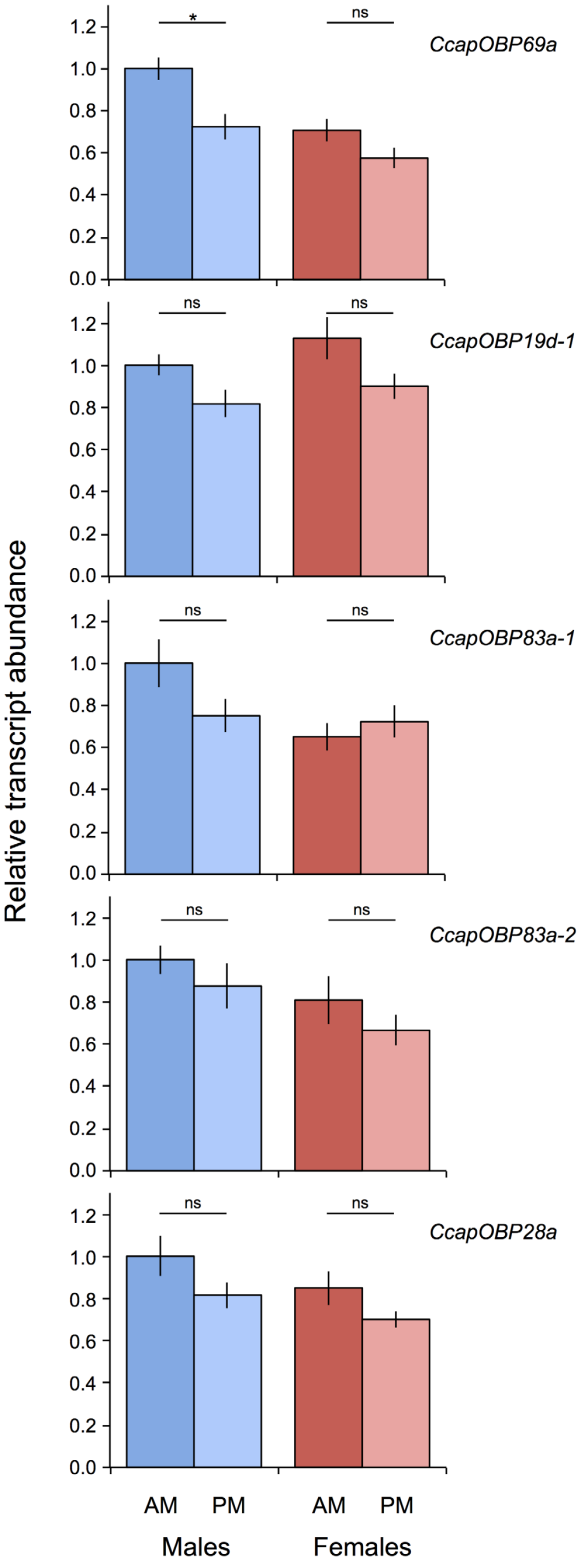
Transcript abundances of five *OBP* genes in the antennae of mature virgin males and females at two time intervals (1–3 and 6–8 hrs after the beginning of the photophase, respectively). Asterisks indicate significant differences in transcript abundances (*P<0.05, unpaired 2-tailed t-tests).

In our insectary conditions, medfly display a bimodal pattern of sexual activity during the day, with one peak at approximately 08∶00–11∶00 hrs and a second minor peak at approximately 13∶00–16∶00 hrs. To evaluate whether the transcriptional activities of the five putative *PBPRP* genes were similar during the two peaks, Real-Time qPCR was performed on RNA from antennae collected from sexually mature virgin individuals of both sexes at 09∶00 and 14∶00. Figure 7 shows that there was a general trend of decreased transcript abundance in the afternoon compared to the morning in both sexes, with the exception of *CcapOBP83a-1* in females. Although these differences may be biologically meaningful, the reduction in transcript levels in the afternoon was statistically significant only for *CcapOBP69a* in male individuals (unpaired t-test, *P = *0.027).

## Discussion

The bioinformatic screening of the three medfly EST libraries derived from head, embryo, testes and male accessory glands [23], [35] revealed 17 transcripts that may be involved in chemosensory perception. Here, through a comparative analysis of their sequence diversity, molecular evolution, and transcription patterns, we provide insights into the possible functional diversification of these genes.

The majority of the 17 medfly sequences pertain to the Classic OBP subfamily, as found previously in all available insect genomes [10], [60]. Also in the medfly, the Classic subfamily display an expansion, represented by the Minus-C group [10]. The Plus-C CcapOBP49a shares much higher similarity to the mosquito Plus-C AaegOBP23 than to any *Drosophila* members of this subfamily.

We used the OBP repertoire of *D. melanogaster* as a reference for a comparative analysis of the medfly OBPs given the close phylogenetic affinity of the two species (80–100 Mya) [61]. Moreover, the olfactory system of *D. melanogaster* is among the best characterized [62]. Apart from *CcapOBP49a*, the medfly genes share high sequence similarity to their *Drosophila* counterparts, with a generally clear 1∶1 orthology. However, in three cases (*CcapOBP19d-1/CcapOBP19d-2, CcapOBP83a-1/CcapOBP83a-2, CcapOBP84a-1/CcapOBP84a-2*), two medfly *OBP* genes correspond to the same *Drosophila* gene. Whether this is the result of duplication and differentiation events during *OBP* evolution in the medfly is a matter for conjecture. It is noteworthy that, in all three cases, both medfly genes display different transcriptional tissue distributions that may also reflect functional divergence.

Several transcripts were present in nearly all the medfly tissue types studied, suggesting functional pleiotrophy. Indeed, their *Drosophila* orthologues, *OBP8a*, *OBP19a*, *OBP19b* and *OBP19d*, occupy transcriptional niches that include genes correlated with olfaction, post-mating behaviour, oviposition and nutrient sensing [63]. Intriguingly, *CcapOBP8a*, like its *Drosophila* orthologue, was also found to be transcribed in the embryo [35], suggesting that it may also be expressed in larval stages where it could be involved in nutrient sensing. In this regard, medfly larvae select feeding sites within the fruit, moving to areas with the highest carbohydrate levels [25], [64].

We suggest that *CcapOBP19d-1*, *CcapOBP28a*, *CcapOBP69a*, *CcapOBP83a-1* and *CcapOBP83a-2* may be implicated in olfactory responses to volatile semiochemicals including those from host plants. This hypothesis is based on the high similarity and close phylogenetic relationships between their predicted protein sequences and *Drosophila* PBPRPs. Moreover, the transcriptional profiles of these genes, although dynamic, suggest transcript enrichment primarly in the antennae. Medfly antennae play a crucial role in intra- and inter-sex communication during courtship behaviour [65]. Males form loose leks on host plant leaves, and perform sexual signaling by emitting a sexual pheromone [24], [25], [30], [66], [67]. Leks increase the overall quantity of pheromone released by the males [68], thus conferring them a selective advantage in terms of female attraction [31], [69]. Receptive mature females visit the leks [70] and choose mates on the basis of their courtship performance, which involves chemical as well as visual and acoustic signals [24], [26]. Medfly females reach sexual maturation two to three days after emergence, and become receptive to the male sexual signaling for copulation [71], [72]. By contrast, males become sexually mature shortly after eclosion [71], [72]. Thus the significant increase in *PBPRP* transcript abundance in four day-old compared to one day-old females may be consequent to the synthesis of the molecular components required for mate recognition. Conversely, one day-old males are already able to mate and, as expected, we do not observe further increases in transcript abundance between one and four day-old males. This is in accordance to what we have previously shown: sexual maturation itself induces profound transcriptional changes in the adult medfly female, and modest variations in the male [36].

After mating, the transcript abundances of the five *PBPRP* genes remained unaltered in females. Given that mated females undergo a dramatic and nearly immediate behavioural switch from male pheromone to host fruit oriented olfactory behaviour for oviposition [73], some alteration in the expression levels of their *PBPRP* genes might have been expected. However, as many components of the male pheromone blend are derived from host plant chemical precursors [74], we cannot exclude that the females employ these PBPRPs to detect volatile host plant emissions for the localization of ripening/ripe fruits suitable for oviposition [74], [75]. By contrast, there was a general reduction in transcript abundance in mated males, significant only for *CcapOBP69a*. As courtship is an extremely energetically costly activity [76], [77], the male may require a period of time to recover after mating and the slight reduction in *PBPRP* transcription may be the result of a reallocation of resources to restore depleted reserves prior to further courtship activity.

Moreover, the decreased, although generally non-significant, *PBPRP* transcript abundance observed during the afternoon peak of male calling (pheromone release), compared to the morning peak, may reflect the reduction in afternoon calling activity observed in our laboratory. It is known that chemoreception, as well as feeding, courtship, mating and oviposition in *Drosophila* undergo circadian regulation [78]–[80]. In this context, it is noteworthy that the genes *OBP83a* and *OBP28a* (orthologues of the medfly *PBPRPs CcapOBP83a-1, CcapOBP83a-2,* and *CcapOBP28a*) are regulated by the CLOCK transcription factor located in the head [81]. Whether also in the medfly the *PBPRP* genes are regulated by diel and/or circadian mechanisms is still an open question and needs to be investigated in a greater detail.

This paper also opens an interesting evolutionary question, i.e. whether different insect lifestyles could be associated with diversification of the chemosensory gene repertoires. The medfly, like other Tephritidae fruit flies including *Bactrocera, Anastrepha* and *Rhagoletis* species, evolved a phytophagous lifestyle in which both the larvae and adults feed on ripening fruit and plant secretions [2], [3]. This represents a major difference with respect to *Drosophila* species [82] and most species within the Tephritoidea superfamily, which are saprophagous, feeding on decaying organic material [2]. These different feeding behaviours are related to adaptive differences in their abilities to perceive, and respond to, different plant volatiles, odours, and pheromone components [3], [83]. In the absence of fully sequenced genomes from other tephritid species, we made a first comparative attempt to assess the presence of similarities/differences between the chemosensory repertoire of *C. capitata* and those of the few tephritid species for which *OBP*/*PBPRP* sequences are available, i.e. *B. dorsalis* [47], *R. suavis* [48] and *R. pomonella* [49]. The most interesting outcome is that the medfly *OBP*/*PBPRP* predicted proteins generally share high amino acid similarity with at least one OBP/PBPRP from the other tephritid species. This is particularly true for the PBPRPs, suggesting a possible functional conservation. This data is important, given that only scattered information are available on the functional roles of OBPs/PBPRPs in these *Bactrocera* and *Rhagoletis* species.

Considering that OBPs/PBPRPs are able to regulate species/sex-specific behaviours related to host/mate location, the knowledge acquired from fruit pests, such as *C. capitata* and other highly invasive tephritids, will aid the development of novel species-specific attractants/repellents for pest control programmes. In this context, the Sterile Insect Technique (SIT), which is a highly efficient environmentally-friendly method employed against invasive tephritid pests [84] will benefit from the development of new effective lures and traps. From the biotechnological point of view, the targeting of PBPRPs is particularly promising, since the identification of molecules able to interact with such chemosensory proteins could provide agents that disrupt mating or oviposition behaviour.

## Supporting Information

Figure S1 Scanning electron microscope image of the head of a female *C. capitata* showing the antennae and maxillary palps.
(TIFF)

Table S1 Primers used in RT-PCR and real time qPCR analyses.
(DOC)

Table S2 BLASTP analyses of *Rhagoletis pomonella* OBPs against the *D. melanogaster* protein database and suggested OBP names.
(DOC)

Table S3 Primers used in RACE and genomic analyses.
(DOC)

Table S4 Similarity between medfly OBPs and those from three other tephritid species, *Bactrocera dorsalis*, *Rhagoletis pomonella* and *Rhagoletis suavis*.
(DOC)
