# Supplementary figures and images for: Sniffing Out Chemosensory Genes from the Mediterranean Fruit Fly, Ceratitis capitata
Source: PLoS One. 2014 Jan 8;9(1):e85523. doi: 10.1371/journal.pone.0085523 (PMC3885724; doi:10.1371/journal.pone.0085523)

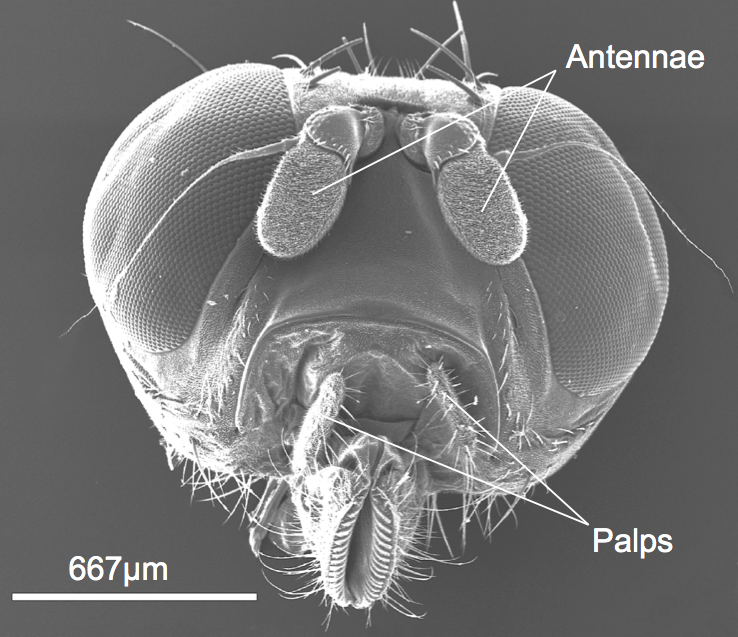

Supplement: Figure S1 — Scanning electron microscope image of the head of a female C. capitata showing the antennae and maxillary palps. (TIFF) [file pone.0085523.s001.tif]
