# Supplementary material for: Sniffing Out Chemosensory Genes from the Mediterranean Fruit Fly, Ceratitis capitata
Source: PLoS One. 2014 Jan 8;9(1):e85523. doi: 10.1371/journal.pone.0085523 (PMC3885724; doi:10.1371/journal.pone.0085523)
Supplement: Table S1 — Primers used in RT-PCR and real time qPCR analyses. (DOC) [file pone.0085523.s002.doc]

Table S1: Primers used in RT-PCR and Real Time qPCR analyses

| **Transcript/Primer** | **Forward primer (5’ - 3’)** | **Reverse primer (5’ - 3’)** | **Expected product (bp)** |
| --- | --- | --- | --- |
| CcapOBP8a | ggaacatatagcggcactt | agcgttgtaacaccactt | 118 |
| CcapOBP19a | atgttgcctagaatgaat | gaacgataccatccttaa | 190 |
| CcapOBP19b | agtaggctttatggcattg | acttcgcacatctcattg | 111 |
| CcapOBP19d-1 | tgatgaatgacgatggcaaaatgg | atcttcagcagcctcgcaatg | 158 |
| CcapOBP19d-2 | ctcgcccaagaatgtaaa | gcaccagccttaatgaat | 188 |
| CcapOBP28a | tgccgctgactccgacattc | tccgcatcgccgtctgtatatg | 174 |
| CcapOBP44a | agccgaacacattgagaa | atatcagccttcacagcat | 183 |
| CcapOBP49a | agttctggaaaggctgtc | atagtatttgttgaggaagtgttt | 122 |
| CcapOBP56d | attcttcacagttgctattg | ccagtctccttgatacac | 126 |
| CcapOBP56h | ttatctcctgtgctgttg | aattctccgatggtgaag | 147 |
| CcapOBP69a | tgtattaaagaaactggcgtcacc | ttctacaatcggcacaaattcagg | 195 |
| CcapOBP83a-1 | ggctttaaatggcagtcgtc | aggccatttatcgtcacgtc | 127 |
| CcapOBP83a-2 | catgaagacggcgaagtg | cagcagcgatgtaaccaa | 146 |
| CcapOBP84a-1 | tttatacgctgctactttgaaa | acagaatccgctgctaat | 101 |
| CcapOBP84a-2 | aattggcaactgaatcgt | gcacttagcaatagcgtaa | 132 |
| CcapOBP99c | tgaggtagttgccattgctg | cactcgatcaccaatcttgc | 118 |
| CcapOBP99d | gctataatgccaagagagt | aaagcccataaatccagat | 121 |
