# Supplementary material for: Sniffing Out Chemosensory Genes from the Mediterranean Fruit Fly, Ceratitis capitata
Source: PLoS One. 2014 Jan 8;9(1):e85523. doi: 10.1371/journal.pone.0085523 (PMC3885724; doi:10.1371/journal.pone.0085523)
Supplement: Table S2 — BLASTP analyses of Rhagoletis pomonella OBPs against the D. melanogaster protein database and suggested OBP names. (DOC) [file pone.0085523.s003.doc]

Table S2 BLASTP analyses of *Rhagoletis pomonella* OBPs against the *D. melanogaster* protein database and suggested OBP names

| Contig | Accession | Best *D. melanogaster* BLASTP hit | e-value | % I/S | Name |
| --- | --- | --- | --- | --- | --- |
| C10486 | EZ126705.1 | OBP19a | 5e-42 | 71/88 | RpomOBP19a |
| C21814 | EZ138033.1 | OBP19b | 1e-28 | 39/61 | RpomOBP19b |
| C21478 | EZ137697.1 | OBP44a | 2e-52 | 63/77 | RpomOBP44a |
| C23516 | EZ139735.1 | OBP56e | 6e-08 | 29/45 | RpomOBP56e |
| C22766 | EZ138985.1 | OBP56h | 5e-16 | 37/51 | RpomOBP56h |
| C20125 | EZ136344.1 | OBP83cd | 8e-29 | 44/66 | RpomOBP83cd |
| C20870 | EZ137089.1 | OBP83ef | 7e-73 | 50/63 | RpomOBP83ef |
| C19484 | EZ135703.1 | OBP99b | 5e-45 | 53/72 | RpomOBP99b |
| C22673 | EZ138892.1 | OBP99c | 1e-52 | 60/79 | RpomOBP99c |
| C23271 | EZ139490.1 | OBP19d | 1e-06 | 28/57 | RpomOBP19d-1 |
| C14712 | EZ130931.1 | OBP19d | 2e-19 | 39/60 | RpomOBP19d-2 |
| C08103 | EZ124322.1 | OBP83a | 8e-48 | 74/86 | RpomOBP83a |
| C22809 | EZ139028.1 | OBP84a | 2e-40 | 54/70 | RpomOBP84a |
| C22963 | EZ139182.1 | OBP28a | 2e-21 | 40/55 | RpomOBP28a-1 |
| C16946 | EZ133165.1 | OBP28a | 2e-15 | 44/71 | RpomOBP28a-2 |
