# Supplementary material for: Sniffing Out Chemosensory Genes from the Mediterranean Fruit Fly, Ceratitis capitata
Source: PLoS One. 2014 Jan 8;9(1):e85523. doi: 10.1371/journal.pone.0085523 (PMC3885724; doi:10.1371/journal.pone.0085523)
Supplement: Table S3 — Primers used in RACE and genomic analyses. (DOC) [file pone.0085523.s004.doc]

Table S3. Primers used in RACE and genomic analyses

|  | **Primer** | **Sequence (5’-3’)** |
| --- | --- | --- |
| 5’ RACE | | |
|  | CcapOBP28a-first5 | ttggtgtgctccattgcgacggatt |
|  | CcapOBP28a-Nested5 | gcaccagcttctccgcgacattcct |
|  | CcapOBP69a-first5 | aatcggcacaaattcaggcggcaat |
|  | CcapOBP69a-Nested5 | gccgtttcacagccatcagctccac |
|  | CcapOBP19d-1-first5 | ttcaacgtcctcatccgttgcacca |
|  | CcapOBP19d-1-Nested5 | cgtcctcatccgttgcaccaacttcg |
|  | CcapOBP83a-1-first5 | tttgagcaccgctggtggaggcca |
|  | CcapOBP83a-1-Nested5 | ttatcgtcacgtcgcggttcctgtg |
|  | CcapOBP83a-2-first5 | ccgactccttccacgagcgatgtaacc |
|  | CcapOBP83a-2-Nested5 | gcgatgttgtgcattgagttcggcaaa |
| 3’ RACE | | |
|  | CcapOBP69a-first3 | aatcggtttgatcgatgcggacaaca |
|  | CcapOBP69a-Nested3 | tattgccgcctgaatttgtgccgatt |
|  | CcapOBP83a-1-first3 | gatgtgagcatccggaaggtgatacgc |
|  | CcapOBP83a-1-Nested3 | cagtgctggaagaaagctgatcctgtgc |
| Genomic Analysis | | |
|  | CcapOBP28a-f | cggctttcaacaaagaggag |
|  | CcapOBP28a-r | cacgtaagtcggtgatcgaa |
|  | CcapOBP69a-f | cgagatgaactacagacggttg |
|  | CcapOBP69a-r | gcttgaaagctgctgcgtat |
|  | CcapOBP19d-1-f | agcagcacagcttttgagaa |
|  | CcapOBP19d-1-r | tcaaaagccagagtcgtcttc |
|  | CcapOBP83a-1-f | cacaggaaccgcgacgtgacgata |
|  | CcapOBP83a-1-r | attgtgttcgtcaatacgcatc |
|  | CcapOBP83a-2-f | ggcaagagcaagcacaattt |
|  | CcapOBP83a-2-r | cacattgtcaaaagcagagtca |
