# Supplementary material for: Sniffing Out Chemosensory Genes from the Mediterranean Fruit Fly, Ceratitis capitata
Source: PLoS One. 2014 Jan 8;9(1):e85523. doi: 10.1371/journal.pone.0085523 (PMC3885724; doi:10.1371/journal.pone.0085523)
Supplement: Table S4 — Similarity between medfly OBPs and those from three other tephritid species, Bactrocera dorsalis, Rhagoletis pomonella and Rhagoletis suavis. (DOC) [file pone.0085523.s005.doc]

Table S4 Similarity between medfly OBPs and those from three other tephritid species, *Bactrocera dorsalis*, *Rhagoletis pomonella* and *Rhagoletis suavis*. BLASTP analyses were performed against the *B. dorsalis* OBP polypeptide sequences present in GenBank and the putative translation products of the *R. pomonella* and *R. suavis* transcript sequences. Short truncated sequences were excluded from the analyses.

| *Ceratitis capitata* | *Bactrocera dorsalis* |  |  | *Rhagoletis pomonella* |  |  | *Rhagoletis suavis* |  |  |
| --- | --- | --- | --- | --- | --- | --- | --- | --- | --- |
| OBP | Putative protein / Accession | e-value | % I/S | Putative protein / Accession | e-value | % I/S | Putative protein / Accession | e-value | % I/S |
|  |  |  |  |  |  |  |  |  |  |
| CcapOBP8a | BdorOBP1 AGS08183.1 | 6e-95 | 80/86 | RpomOBP99c EZ138892.1 | 4e-17 | 28/50 | RsuaOBP5 EX453824 | 1e-63 | 79/84 |
|  | BdorOBP10 AGS08192.1 | 2e-17 | 22/48 | RpomOBP44a EZ137697.1 | 1e-14 | 23/53 |  |  |  |
|  | BdorOBP7 AGS08189.1 | 2e-11 | 25/45 | RpomOBP99b EZ135703.1 | 4e-13 | 26/49 |  |  |  |
|  | BdorOPB9 AGS08191.1 | 1e-10 | 24/41 |  |  |  |  |  |  |
|  |  |  |  |  |  |  |  |  |  |
| CcapOBP19a | - | - | - | RpomOBP19a EZ126705.1 | 2e-55 | 71/81 | RsuaOBP3 EX453821 | 6e-06 | 31/52 |
|  |  |  |  | RpomOBP83a EZ124322.1 | 4e-06 | 31/52 |  |  |  |
|  |  |  |  | RpomOBP44a EZ137697.1 | 6e-06 | 25/37 |  |  |  |
|  |  |  |  |  |  |  |  |  |  |
| CcapOBP19b | - | - | - | RpomOBP19b EZ138033.1 | 1e-65 | 65/77 | RsuaOBP9 EX453831 | 7e-08 | 28/47 |
|  |  |  |  | RpomOBP19d-1 EZ139490.1 | 3e-08 | 23/51 | RsuaOBP7 EX453827 | 5e-06 | 30/48 |
|  |  |  |  |  |  |  |  |  |  |
| CcapOBP19d-1 | BdorOBP4 AGS08186.1 | 2e-06 | 23/46 | RpomOBP19d-2 EZ130931.1 | 9e-53 | 67/83 | RsuaOBP7 EX453827 | 7e-69 | 68/81 |
|  |  |  |  | RpomOBP28a-1 EZ139182.1 | 6e-37 | 41/62 | RsuaOBP8 EX453830 | 8e-38 | 42/64 |
|  |  |  |  | RpomOBP28a-2 EZ133165.1 | 1e-15 | 50/70 | RsuaOBP9 EX453831 | 8e-25 | 40/58 |
|  |  |  |  | RpomOBP19d-1 EZ139490.1 | 7e-08 | 24/48 |  |  |  |
|  |  |  |  |  |  |  |  |  |  |
| CcapOBP19d-2 | BdorOBP4 AGS08186.1 | 3e-06 | 39/53 | RpomOBP28a-1 EZ139182.1 | 9e-36 | 42/58 | RsuaOBP8 EX453830 | 1e-35 | 41/58 |
|  |  |  |  | RpomOBP19d-2 EZ130931.1 | 3e-12 | 25/48 | RsuaOBP7 EX453827 | 1e-21 | 29/52 |
|  |  |  |  | RpomOBP28a-2 EZ133165.1 | 3e-10 | 38/63 | RsuaOBP9 EX453831 | 2e-16 | 32/50 |
|  |  |  |  | RpomOBP19d-1 EZ139490.1 | 9e-06 | 27/47 |  |  |  |
|  |  |  |  |  |  |  |  |  |  |
| CcapOBP28a | BdorOBP5 AGS08187.1 | 3e-08 | 30/43 | RpomOBP28a-2 EZ133165.1 | 7e-45 | 76/86 | RsuaOBP9 EX453831 | 3e-82 | 75/85 |
|  |  |  |  | RpomOBP28a-1 EZ139182.1 | 1e-26 | 30/55 | RsuaOBP7 EX453827 | 8e-29 | 35/52 |
|  |  |  |  | RpomOBP19d-2 EZ130931.1 | 7e-20 | 38/52 | RsuaOBP8 EX453830 | 2e-26 | 30/54 |
|  |  |  |  |  |  |  |  |  |  |
| CcapOBP44a | BdorOPB9 AGS08191.1 | 2e-39 | 42/62 | RpomOBP44a EZ137697.1 | 2e-89 | 95/97 | RsuaOBP5 EX453824 | 5e-10 | 25/53 |
|  | BdorOBP7 AGS08189.1 | 3e-31 | 37/57 | RpomOBP99b EZ135703.1 | 4e-31 | 37/57 |  |  |  |
|  | BdorOBP10 AGS08192.1 | 4e-23 | 35/56 | RpomOBP99c EZ138892.1 | 2e-24 | 36/56 |  |  |  |
|  | BdorOBP1 AGS08183.1 | 3e-20 | 28/55 | RpomOBP83ef EZ137089.1 | 8e-12 | 28/44 |  |  |  |
|  | BdorOBP8 AGS08190.1 | 3e-10 | 24/44 |  |  |  |  |  |  |
|  |  |  |  |  |  |  |  |  |  |
| CcapOBP49a | - | - | - | - | - | - | - | - | - |
|  |  |  |  |  |  |  |  |  |  |
| CcapOBP56d | BdorOBP3 AGS08185.1 | 3e-57 | 66/82 | RpomOBP56e EZ139735.1 | 1e-13 | 31/52 | RsuaOBP1 EX453819 | 2e-16 | 31/45 |
|  | BdorOBP4 AGS08186.1 | 5e-56 | 62/81 |  |  |  | RsuaOBP2 EX453820.1 | 8e-09 | 25/50 |
|  | BdorOBP2 AGS08184.1 | 3e-50 | 67/83 |  |  |  | RsuaOBP3 EX453821 | 3e-08 | 26/49 |
|  | BdorOBP2* AGO28153.1 | 1e-11 | 28/45 |  |  |  | RsuaOBP8 EX453830 | 5e-06 | 25/44 |
|  | BdorOBP5 AGS08187.1 | 1e-08 | 27/47 |  |  |  |  |  |  |
|  | BdorOBP10 AGS08192.1 | 1e-06 | 30/47 |  |  |  |  |  |  |
|  |  |  |  |  |  |  |  |  |  |
| CcapOBP56h | BdorOBP5 AGS08187.1 | 2e-57 | 63/81 | RpomOBP56h EZ138985.1 | 2e-55 | 64/83 | RsuaOBP6 EX453825 | 2e-53 | 68/84 |
|  | BdorOBP6 AGS08188.1 | 4e-11 | 32/51 | RpomOBP19d-1 EZ139490.1 | 6e-09 | 23/41 | RsuaOBP1 EX453819 | 5e-09 | 28/43 |
|  | BdorOBP4 AGS08186.1 | 5e-10 | 22/44 |  |  |  | RsuaOBP2 EX453820.1 | 3e-07 | 25/42 |
|  | BdorOBP2* AGO28153.1 | 2e-08 | 27/44 |  |  |  | RsuaOBP3 EX453821 | 7e-07 | 26/45 |
|  | BdorOBP2 AGS08184.1 | 6e-08 | 27/45 |  |  |  | RsuaOBP9 EX453831 | 6e-06 | 31/53 |
|  | BdorOBP3 AGS08185.1 | 4e-06 | 23/45 |  |  |  |  |  |  |
|  |  |  |  |  |  |  |  |  |  |
| CcapOBP69a | BdorOBP2* AGO28153.1 | 2e-15 | 27/52 | RpomOBP83a EZ124322.1 | 8e-11 | 33/66 | RsuaOBP1 EX453819 | 5e-78 | 69/85 |
|  | BdorOBP4 AGS08186.1 | 4e-11 | 24/46 | RpomOBP56h EZ138985.1 | 1e-09 | 32/45 | RsuaOBP2 EX453820.1 | 1e-16 | 29/52 |
|  | BdorOPB9 AGS08191.1 | 2e-09 | 28/44 | RpomOBP28a-1 EZ139182.1 | 2e-09 | 29/43 | RsuaOBP3 EX453821 | 4e-14 | 28/53 |
|  | BdorOBP2 AGS08184.1 | 2e-09 | 26/45 | RpomOBP99b EZ135703.1 | 1e-08 | 22/46 | RsuaOBP6 EX453825 | 3e-11 | 30/46 |
|  | BdorOBP3 AGS08185.1 | 1e-08 | 29/43 |  |  |  | RsuaOBP8 EX453830 | 6e-10 | 28/43 |
|  | BdorOBP5 AGS08187.1 | 4e-06 | 27/45 |  |  |  |  |  |  |
|  |  |  |  |  |  |  |  |  |  |
| CcapOBP83a-1 | BdorOBP2* AGO28153.1 | 4e-50 | 50/70 | RpomOBP83a EZ124322.1 | 1e-73 | 83/89 | RsuaOBP3 EX453821 | 9e-96 | 85/90 |
|  | BdorOBP4 AGS08186.1 | 1e-09 | 25/45 | RpomOBP99b EZ135703.1 | 9e-08 | 24/50 | RsuaOBP2 EX453820.1 | 2e-55 | 49/73 |
|  | BdorOBP3 AGS08185.1 | 4e-09 | 27/51 | RpomOBP56h EZ138985.1 | 2e-06 | 24/43 | RsuaOBP1 EX453819 | 2e-12 | 25/47 |
|  | BdorOBP2 AGS08184.1 | 2e-08 | 27/49 |  |  |  | RsuaOBP6 EX453825 | 1e-07 | 24/43 |
|  | BdorOBP10 AGS08192.1 | 5e-06 | 22/49 |  |  |  |  |  |  |
|  |  |  |  |  |  |  |  |  |  |
| CcapOBP83a-2 | BdorOBP2* AGO28153.1 | 3e-94 | 84/91 | RpomOBP83a EZ124322.1 | 2e-38 | 55/75 | RsuaOBP2 EX453820.1 | 2e-93 | 82/91 |
|  | BdorOBP4 AGS08186.1 | 6e-11 | 27/53 | RpomOBP99b EZ135703.1 | 5e-06 | 33/58 | RsuaOBP3 EX453821 | 4e-54 | 56/78 |
|  | BdorOBP2 AGS08184.1 | 4e-10 | 29/52 |  |  |  | RsuaOBP1 EX453819 | 2e-12 | 25/48 |
|  | BdorOBP3 AGS08185.1 | 1e-08 | 26/50 |  |  |  | RsuaOBP6 EX453825 | 2e-06 | 23/44 |
|  |  |  |  |  |  |  |  |  |  |
| CcapOBP84a-1 | BdorOPB9 AGS08191.1 | 2e-06 | 34/50 | RpomOBP84a EZ139028.1 | 3e-40 | 52/70 | RsuaOBP4 EX453822 | 7e-41 | 40/57 |
|  |  |  |  |  |  |  |  |  |  |
| CcapOBP84a-2 | - | - | - | RpomOBP84a EZ139028.1 | 5e-75 | 80/87 | RsuaOBP4 EX453822 | 3e-82 | 70/81 |
|  |  |  |  |  |  |  |  |  |  |
| CcapOBP99c | BdorOBP10 AGS08192.1 | 3e-91 | 81/91 | RpomOBP99c EZ138892.1 | 4e-89 | 79/90 | RsuaOBP5 EX453824 | 2e-14 | 26/54 |
|  | BdorOBP1 AGS08183.1 | 1e-20 | 26/50 | RpomOBP44a EZ137697.1 | 7e-22 | 32/54 |  |  |  |
|  | BdorOPB9 AGS08191.1 | 1e-19 | 32/53 | RpomOBP99b EZ135703.1 | 2e-16 | 27/51 |  |  |  |
|  | BdorOBP7 AGS08189.1 | 7e-14 | 26/49 |  |  |  |  |  |  |
|  |  |  |  |  |  |  |  |  |  |
| CcapOBP99d | BdorOBP10 AGS08192.1 | 1e-13 | 33/55 | RpomOBP99c EZ138892.1 | 1e-13 | 33/55 | RsuaOBP5 EX453824 | 5e-12 | 25/49 |
|  | BdorOBP1 AGS08183.1 | 2e-12 | 27/51 |  |  |  |  |  |  |
|  |  |  |  |  |  |  |  |  |  |

* Unpublished BdorOBP2 available in GenBank
